# Impact of Mobile Apps in Conjunction With Percutaneous Endoscopic Gastrostomy on Patients' Complications, Quality of Life, and Health-Related Self-Care Behaviors: Randomized Clinical Trial

**DOI:** 10.2196/48970

**Published:** 2023-10-20

**Authors:** Bi-Lian Chen, Han-Chung Lien, Shyh-Sheng Yang, Shiao-Chi Wu, Hsien-Hsien Chiang, Li-Chan Lin

**Affiliations:** 1 Department of Nursing National Yang Ming Chiao Tung University Taipei City Taiwan; 2 Department of Nursing Taichung Veterans General Hospital Taichung Taiwan; 3 Division of Gastroenterology Taichung Veterans General Hospital Taichung Taiwan; 4 The Division of Thoracic Surgery Taichung Veterans General Hospital Taichung Taiwan; 5 Institute of Health and Welfare Policy National Yang Ming Chiao Tung University Taipei City Taiwan; 6 Department of Nursing Asia University Taichung Taiwan

**Keywords:** percutaneous endoscopic gastrostomy, mobile applications tracking system, self-care, complications, quality of life, mobile application, mHealth app, mHealth intervention, health promotion, health education, endoscopy, application, education, gastrostomy, care, prevention, behavior, tracking, utilization

## Abstract

**Background:**

Percutaneous endoscopic gastrostomy (PEG) is commonly chosen for long-term enteral nutrition support. However, common complications of PEG include wound infection, leakage, obstruction, bleeding, dislodgement, pneumonia, peritonitis, and more. The anticipation of these complications by both patients and their family caregivers underscores the essential requirement of ongoing technical guidance for the daily care of PEG and the adoption of preventative strategies.

**Objective:**

This study aimed to establish and compare a health education program utilizing a tracking system for PEG using a mobile app (PEG app) and instant messaging software versus a paper-based health education program with instant messaging software. Their effectiveness in preventing complications, avoiding hospital readmissions, improving self-care practices, and enhancing quality of life outcomes was assessed.

**Methods:**

A randomized controlled trial design was used, and the study sample consisted of patients from a medical center in central Taiwan who underwent thoracic surgery or gastroenterology procedures. Inclusion criteria were being a new case undergoing his or her first gastric tube insertion and having the ability to operate a smartphone. Exclusion criteria were cases requiring tube replacement or nasogastric tubes. A total of 74 participants were enrolled, with 37 participants in the experimental group and 37 participants in the control group. Data collection took place from hospitalization until 1 month after discharge. The experimental group received care using the gastric tube tracking system (PEG app) and the Line app that included phone, text, and photo capture capabilities, while the control group received routine nursing care and used the Line app.

**Results:**

The experimental group demonstrated a significant reduction in the occurrence of complications compared with the control group (*χ^2^*_1_=12.087, *P*=.001). Specifically, the occurrence of leakage events was significantly lower in the experimental group than in the control group (*χ^2^*_1_=12.906, *P*=.001). However, the experimental group exhibited superior self-care ability compared with the control group (*t*_72_=2.203, *P*=.03). There was no significant difference in overall quality of life scores between the experimental and control groups (*t*_72_=1.603, *P*=.11). However, the experimental group showed better social aspects of quality of life than the control group (*t*_72_=2.164, *P*=.03).

**Conclusions:**

Integration of the PEG app with instant messaging can enhance self-care ability, improve social aspects of quality of life, and reduce complications. The study results suggest that the PEG app could be used as an adjunct tool to promote patients’ self-directed management of their gastric tube at home, particularly for patients who have undergone their first PEG placement and are being discharged from the hospital.

**Trial Registration:**

Chinese Clinical Trial Registry ChiCTR2300071271; https://tinyurl.com/4vvy584e

## Introduction

Percutaneous endoscopic gastrostomy (PEG) is a preferred method for long-term enteral nutrition support. Studies indicate that 5% to 7.8% of nursing home residents receive gastrostomy feeding [1-3]. Wong et al [4] found that 2.5% of residents in Singapore’s long-term care facilities were using a gastrostomy tube. In Taiwan, cultural factors and the lack of reimbursement for PEG procedures by the National Health Insurance before 2009 contributed to a reported prevalence rate of 0.4% for PEG use, with percutaneous endoscopic jejunostomy use in long-term care facilities ranging from 0.1% to 0.3% [5]. Although these figures suggest a lower prevalence in Asian countries compared with Western countries, it is conceivable that there will be an increased use of PEG for long-term enteral feeding in the near future. This prediction is based on the availability of health education and coverage for PEG insertion by the National Health Insurance in Taiwan as of 2009.

Common complications of PEG include wound infection (3%-50%), leakage (10%-42.3%), obstruction (8%-35%), bleeding (32%), dislodgement (14.3%), pneumonia, peritonitis (1%-18%), and buried bumper syndrome (1.5%-0.8%) [6-10]. A study by Ang et al [11] that involved interviews of 18 patients and caregivers about their experience with PEG tube use revealed that home-based patients with PEG and their caregivers often feel anxious about insufficient self-care knowledge and information about what to expect after tube insertion. They particularly fear the occurrence of complications. Farrag et al [12] also noted that handling the complexities of enteral feeding techniques requires ongoing technical guidance from someone to explain the daily care of PEG and how to avoid complications, as well as to observe signs of complications and promptly report them to health care personnel for appropriate management.

With the advancement of technology and the widespread use of smartphones, instant messaging apps, which can be downloaded for free on smartphones or computers via the internet, have transformed health education from static paper-based methods to dynamic audiovisual and video formats. These apps provide real-time voice and visual information, allowing one-on-one or group video chats; the exchange of photos, audio, and video; and real-time online question and answer functionality. However, despite the rapidity and convenience of instant messaging as a means of communication, when applied to disease prevention and health education campaigns, there is often a lack of standardized guidelines within the medical field [13-17].

As a result, medical apps, including medical informatics apps, have emerged as a new educational approach. These apps offer advantages of standardization, privacy, data confidentiality, and systematic management [18]. The National Institute for Health and Care Excellence in the United Kingdom defines mobile health (mHealth) as the use of software downloaded on smartphones or computers via the internet for the exchange of calls as well as voice, text, and image messages [19,20]. Health care providers can use mHealth to track, monitor, and analyze patients’ health data in real time, providing feedback on their health status [21]. This approach can improve patient compliance, strengthen patient-centered self-management of health [22], reduce emergency room visits [23], and decrease health care expenses resulting from complications [24,25].

These effects result from integrating smartphone apps with technology that changes people’s lifestyles and behavior [26]. The support by Wong et al [27-29] for the development of mHealth apps, particularly those grounded in a health-social approach model, underscores their potential to improve self-care, quality of life, and health outcomes for community-dwelling older adults and patients with chronic diseases. These apps may achieve this by enhancing self-efficacy, promoting sustained behavior change, encouraging regular app use, leveraging visual communication, and reducing reliance on traditional health care services.

Medical information apps have demonstrated effectiveness for privacy and confidentiality, self-health management, and enhancing self-care capabilities. We conducted a systematic literature review with specific search criteria combining the keywords “percutaneous endoscopic gastrostomy” and “mHealth application” using the Boolean operator “AND” and restricting the publication year range from 1995 to 2023. These criteria were applied to the following 4 databases: CINAHL, ProQuest, PubMed, and the Cochrane Library. Among the databases scrutinized, only ProQuest yielded 2 conference abstracts that were relevant to the convergence of PEG and mHealth apps within the specified publication year range. The literature search failed to uncover any articles specifically addressing the use of mHealth apps in the context of PEG procedures.

As such, the aim of this study was to establish and compare the effectiveness of a mobile app for PEG integrated with real-time communication software with that of a paper-based health education program also integrated with real-time communication software. This study evaluated the implications on several key health-related outcomes, including complications and self-care, as well as perceived well-being in terms of quality of life. Additionally, the study assessed the impact on the use of health care services, specifically looking at hospital or emergency readmissions.

## Methods

### Study Design

**Figure 1:**
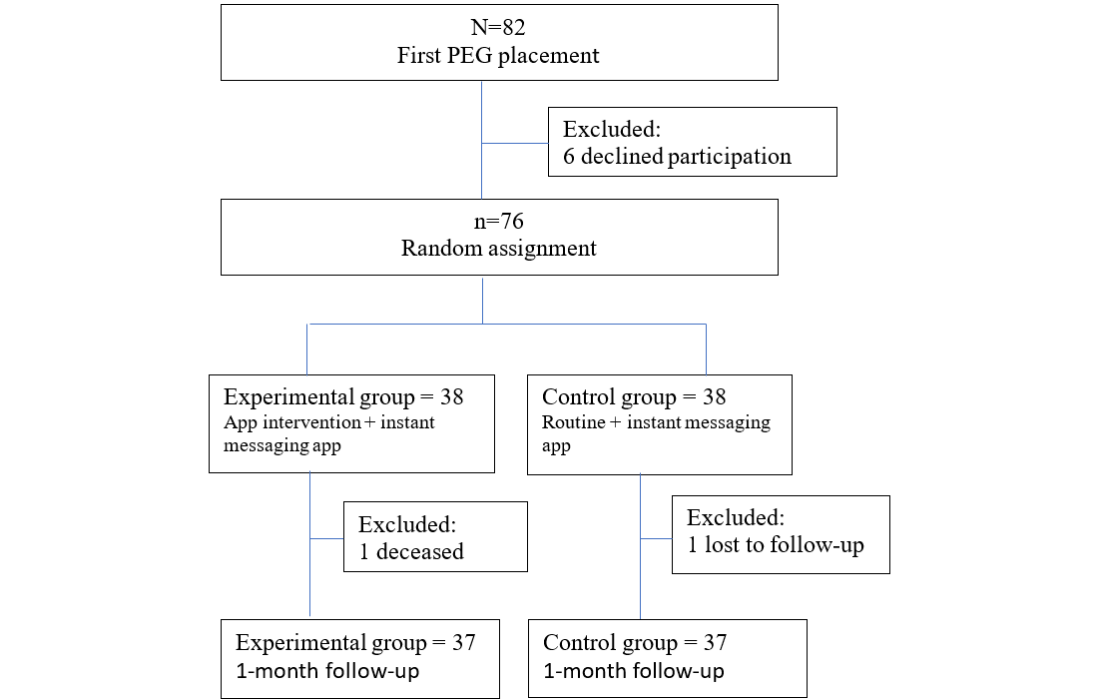
Flow of participants for the study of an app intervention for PEG. PEG: percutaneous endoscopic gastrostomy.

This study used a randomized controlled trial design with a study period spanning from August 7, 2018, to December 31, 2022. Random allocation was achieved using computer-generated grouping, with numbers assigned and placed in sealed envelopes. Envelopes were drawn by cases at the time of enrollment to determine group allocation, continuing until the estimated sample size was reached, as illustrated in Figure 1.

### Recruitment

A total of 82 patients underwent initial catheter placement. After excluding 6 patients who refused participation, 76 patients were randomized and assigned to respective groups, with 38 patients in each group for the study. During the 1-month follow-up period, there was 1 death owing to complications of chemotherapy in the experimental group, and 1 patient was lost to follow-up in the control group, resulting in 37 patients in each group who completed the study.

### Research Site and Participants

The study was conducted at a prestigious medical center located in the central region of Taiwan. Inclusion criteria for the study were as follows: (1) patients who were undergoing their first PEG placement; (2) patients who had access to mobile devices such as smartphones, tablets, or computers; (3) patients or their caregivers who were able to operate mobile devices; (4) patients who could upload data according to the designated schedule; and (5) patients who could communicate in English or Taiwanese. Exclusion criteria were as follows: (1) patients with nasogastric tubes and (2) patients who required replacement of gastrostomy or nasogastric tubes during the study period.

### Sample Size Estimation for Power Calculation

The sample size estimation for power calculation was grounded in the primary outcome measure, which focused on the most common complication—wound infection. This approach was inspired by the work of Pattison and Young [30], who indicated that the infection rates were 31% in the context of an educational booklet intervention and 58% in the control group. In the Department of General Surgery at the University of Washington, the readmission rate because of postoperative wound infection within 1 month after an intervention with a mobile app was 38% (5/13), according to Sanger et al [31]. According to the Power and Sample Size Program, sample size estimation was performed using the occurrence of wound infection complications as the outcome indicator for both the experimental and control groups treated as independent samples. With expected rates of 38% in the experimental group and 58% in the control group, an α level of .05, and a β level of .9, the calculated sample size was 32 patients in each group, for a total of 64 patients. Considering an estimated loss to follow-up rate of 10 patients (15%), a total of 74 patients were planned to be enrolled in the study.

### Randomization and Groups

The research tool involves the completion of a “Participant Consent Form” by the patient or their legal representative, followed by random allocation to either the intervention group or the control group through a drawing of lots. Patients in the intervention group had the mobile device app “Percutaneous Endoscopic Gastrostomy Tracking System (HTML 5)“ (PEG app) installed during their hospitalization.

The intervention group received the PEG app and Line app (for instant messaging) for guidance. The protocol encompassed a teaching and feedback process administered by the principal investigator. This process involved daily changes of the wound dressing and applying sterile gauze, which were conducted by the patient or their caregiver postoperation. Additionally, the process included ongoing monitoring of the wound's condition and regular measurement of body temperature and body weight for a duration of 1 week. Patients were instructed to self-report any symptoms related to their wound, including but not limited to redness, swelling, heat, pain, discharge, and odor, using the designated mobile app. They were encouraged to upload daily wound photos until the completion of 1 week after the procedure. In addition to the aforementioned wound symptom reporting, patients were asked to complete a self-care knowledge questionnaire as a pretest. Furthermore, in the fourth week following tube placement, patients were instructed to complete questionnaires related to self-care knowledge, self-care ability, and their quality of life through the designated mobile app. In the event of an emergency, such as visits to the emergency department within 3 days or hospital readmissions within 14 days, patients or their family members were instructed to use the mobile app to report the situation on a daily basis. The data collected through the app were then accessed by the principal investigator through a designated website. The Line app (instant messaging) provided real-time communication for patients or family members to address any caregiving issues, including the ability to conduct voice or video calls and upload wound photos for immediate response.

The control group received traditional paper-based gastric tube education handouts, which include information on the purpose, indications, daily care instructions, and complications. Additionally, for ethical consideration, the Line app (instant messaging) was used for real-time communication with patients or family members to address any caregiving issues, including immediate response through voice or video calls and uploading wound photos for prompt evaluation.

### Research Instruments and Assessment of Reliability and Validity

Regarding hardware, the mobile devices included smartphones, tablets, computers, a server, a thermometer, and a scale. Regarding software, the PEG tracking system was compatible with Android or iOS mobile devices or desktop computers with the Windows operating system, and the Line app allowed real-time communication. The research team installed the ”Active Server Pages (the Gastric Tube Monitoring Platform)“ through the Google browser, which automatically sent alerts to the platform regarding patients’ ”elevated body temperature“ and ”signs of wound infection.“ The assessment tools included basic patient information, physiological monitoring forms, wound assessment forms and complication reporting, self-care knowledge, self-care ability, and quality of life measures.

The app interface (prototype) was designed based on a needs analysis of patients, family members, and clinical nurses. The researchers used draw.io software to create the interface, which included the login page, the home page, basic information, wound inspection form, temperature trend chart, gastric tube wound photography, recognition of complications, complications reporting, question-and-answer functionality, quality of life questionnaire, self-care knowledge questionnaire, and self-care ability questionnaire.

### Outcome Measures

Data collection took place at 2 specific time points: initially, before the intervention began to gather demographic information and assess self-care knowledge. Subsequently, data collection occurred after the intervention, within 1 week of the completion of the 1-month study period, to assess complications, quality of life, self-care knowledge, and self-care ability.

#### Primary Outcomes

Following the health-social approach model, complications and quality of life were the primary outcome measures.

##### Complications

Complications including wound infection, leakage, migration, pneumonia, granulation, bleeding, admission to the emergency department within 3 days, and hospital readmission within 14 days were assessed.

##### World Health Organization Quality of Life Brief Version

In 1995, the World Health Organization (WHO) established a quality of life questionnaire (WHOQOL) that includes the following 4 major domains: psychological, physiological, social, and environmental. The domains use a 5-point Likert scale. A score of 1 represents ”very dissatisfied,“ 2 represents ”dissatisfied,“ 3 represents ”neutral,“ 4 represents ”satisfied,“ and 5 represents ”very satisfied“ [32]. The Taiwan version of the WHOQOL questionnaire is a concise version, with 26 items and 2 additional local items, totaling 28 items (WHOQOL-BREF). Translated by Yao et al [33], the questionnaire has shown good internal consistency and reliability, with a Cronbach α of .91 and content validity ranging from 0.53 to 0.78.

#### Secondary Outcomes

##### Self-Care Knowledge Assessment

The self-care knowledge assessment primarily evaluated the level of understanding by individuals or primary caregivers about self-care for gastric tubes, based on the following 4 dimensions: wound care, tube feeding, complications, and emergency medical care. A self-designed questionnaire with 10 items assessed self-care knowledge, with correct answers given a score of 1 and incorrect or unknown answers given a score of 0. A higher score indicates better self-care knowledge, with a total possible score of 10 and a minimum score of 0. The content validity index (CVI) during the first round of expert review was 0.58. Following semantic modifications to the eighth item, “emergency situations related to gastric tubes,” to remove compound options, as suggested by the experts, the second round of expert review yielded a CVI of 1. The internal consistency of the ”self-care knowledge scale“ was determined to be Cronbach α=.66.

##### Self-Care Ability

The self-designed questionnaire for self-care ability primarily assessed the level of competence in performing gastric tube care behaviors by patients or primary caregivers. It covers the following 3 dimensions: measures for tube feeding, prevention of complications, and observation of symptoms of complications. The self-designed questionnaire consists of 11 items, which are rated using a 5-point Likert scale, with response options of ”always“ (5 points), ”often“ (4 points), ”sometimes“ (3 points), ”rarely“ (2 points), and ”never“ (1 point). After revisions, the second round of expert review yielded a CVI of 0.98. The internal consistency reliability of the ”self-care ability scale“ was Cronbach α=.78.

### Ethical Considerations

This study was approved by the Human Research Ethics Review Committee of Taichung Veterans General Hospital (on August 7, 2018; institutional review board number: CF18189A-1).

### Data Processing and Statistical Analysis

Descriptive analysis was performed using SPSS 26.0, while inferential statistical analysis involved chi-square tests, independent *t* tests, and paired *t* tests to analyze categorical or continuous variables to examine whether there were differences between the 2 groups being compared.

## Results

The mean ages of the experimental and control groups were 60.63 (SD 10.29) years and 60.79 (SD 9.63) years, respectively. In the experimental group, there were 35 men (35/38, 92%); in the control group, there were 31 men (31/38, 82%). There were no significant differences between the 2 groups regarding basic demographic characteristics, as shown in Table 1.

In addition to demographic data, self-care knowledge was the sole research variable that had a pretest assessment. During the study, 1 participant passed away, and another was lost to follow-up, accounting for a small proportion of missing data (2/76, 3%). To address this limited missing data, imputation was conducted by utilizing the mean self-care knowledge in the posttest for both the experimental and control groups. Table 2 shows that the posttest knowledge scores in the experimental group and control group were significantly higher than the pretest scores, with significant differences observed in the paired *t* tests (experimental group: t_37_=3.99, *P*=.001; control group: t_37_=4.75, *P*<.001). Upon further analysis of the change in self-care knowledge scores from pre to postintervention, the experimental group exhibited a larger change in scores than the control group. However, it is important to note that this difference did not reach statistical significance.

Table 3 shows that 5 individuals in the experimental group and 19 individuals in the control group experienced complications. The incidence of complications was significantly lower in the experimental group than in the control group (*χ^2^*_1_=12.087, *P*=.001). Further analysis revealed that the control group had a higher incidence of complications, including infection, leakage, displacement, pneumonia, bleeding, and granulation tissue formation, than the experimental group. However, only the incidence of leakage was significantly lower in the experimental group than in the control group (*χ^2^*_1_=12.906, *P*=.001). In addition, there were no significant differences between the 2 groups regarding visits to the emergency department within 3 days and hospital readmission within 14 days.

In terms of self-care ability, the experimental group demonstrated significantly better self-care ability than the control group (t_72_=2.203, *P*=.03), as indicated by higher scores on the self-care ability scale. Higher scores on the scale represent better self-care ability.

In terms of quality of life, there were no significant differences in total scores between the experimental and control groups. However, upon further examination of the quality of life in terms of physiological, psychological, social, and environmental aspects, the experimental group showed significantly higher scores in the social domain than the control group (t_72_=2.164, *P*=.03). At the same time, there were no differences in other domains, as shown in Table 4.

**Table 1:** Participant characteristics (N=76).

Characteristics		Control group (n=38)	Experimental group (n=38)	Statistical test (*t* test or chi-square test; *df*)	*P* value
Age (years), mean (SD)		60.79 (9.63)	60.63 (10.29)	0.06 (74)^a^	.95
BMI (kg/m^2^), mean (SD)		21.04 (4.19)	21.93 (4.24)	0.92 (74)^a^	.36
**Sex, n (%)**				1.84 (1)^b^	.18
	Female	7 (18)	3 (8)		
	Male	31 (82)	35 (92)		
Chemotherapy, n (%)		33 (87)	34 (90)	0.126 (1)^b^	.72
Radiotherapy, n (%)		34 (90)	34 (90)	0 (1)^b^	>.99
**Caregiving responsibility, n (%)**				4.41 (2)^b^	.11
	Spouse	24 (63)	15 (40)		
	Child or grandchild	10 (26)	15 (40)		
	Other	4 (11)	8 (21)		
**Patient education, n (%)**				2.65 (4)^b^	.62
	Illiterate	2 (5)	1 (3)		
	Primary school	5 (13)	7 (18)		
	Junior high school	11 (29)	7 (18)		
	Senior high school	20 (53)	22 (58)		
	Undergraduate or higher	0 (0)	1 (3)		
**Caregiver education (n=71), n (%)**				6.62 (5)^b^	.20
	Illiterate	1 (3)	0 (0)		
	Primary school	2 (5)	0 (0)		
	Junior high school	5 (14)	2 (6)		
	Senior high school	17 (46)	14 (41)		
	Undergraduate	11 (30)	13 (38)		
	Graduate school or higher	1 (3)	5 (15)		
NG^c^ used, n (%)		12 (32)	12 (32)	0 (1)^b^	>.99

^a^*t* test.

^b^Chi-square test.

^c^NG: nasogastric.

**Table 2:** Comparison of knowledge between pre and posttests for the experimental and control groups (N=76).

Group^a^	Pretest score	Posttest score	MD^b^	Paired *t* test (*df*)	*P* value
Control group	7.03 (1.61)	8.29 (1.22)	1.26	4.75 (37)	<.001
Experimental group	7.08 (1.53)	8.61 (1.79)	1.52	3.99 (37)	.001

^a^Comparison between the groups: t_74_=0.81, *P*=.42.

^b^MD: mean difference.

**Table 3:** Comparison of complications between the experimental and control groups (N=74).

Complications	Control group (n=37), n (%)	Experimental group (n=37), n (%)	Chi-square (*df*)	*P* value
Complication of any type (n=24)	19 (51)	5 (14)	12.09 (1)	.001
Infection	7 (19)	3 (8)	1.85 (1)	.17
Leakage	15 (41)	2 (5)	12.91 (1)	.001
Migration	1 (3)	0 (0)	—^a^	>.99
Pneumonia	1 (3)	0 (0)	—^a^	>.99
Granulation	3 (8)	0 (0)	—^a^	>.99
Bleeding	1 (3)	0 (0)	—^a^	>.99
Admission to the emergency department (3 days)	1 (3)	0 (0)	—^a^	>.99
Hospital readmission (14 days)	2 (5)	2 (5)	—^a^	>.99

^a^Not applicable because Fisher exact tests were conducted.

**Table 4:** Comparison of self-care ability and quality of life (QOL) between the experimental and control groups (N=74), assessed using independent *t* tests.

Self-care ability and quality of life			Control group (n=37), mean (SD)		Experimental group (n=37), mean (SD)		*t* test (*df*)		*P* value
Ability for self-care of the PEG^a^			47.49 (5.63)		50.30 (5.33)		2.200 (72)		.03
**QOL**									
	Physical	25.27 (5.69)		26.27 (4.50)		0.837 (72)		.41	
	Psychological	19.51 (3.71)		20.57 (3.67)		1.220 (72)		.22	
	Social	33.05 (6.70)		35.97 (4.72)		2.164 (72)		.03	
	Environment	14.11 (2.72)		14.76 (2.37)		0.567 (72)		.28	
	Total QOL score	91.95 (16.81)		97.57 (13.12)		1.603 (72)		.11	

^a^PEG: percutaneous endoscopic gastrostomy.

## Discussion

### Principal Findings

The research findings demonstrated that using the PEG and Line apps can improve self-care behaviors, reduce complications, and enhance quality of life at the social level. The experimental group demonstrated significantly better self-care abilities than the control group. In terms of behavior change and habit formation, the experimental group utilized the app platform to proactively capture and report abnormal wound information, such as redness, swelling, heat, and pain, through daily wound photos, wound infection checks, and vital sign data feedback. Researchers received email notifications and were able to immediately observe changes in the patients' recent wound condition, particularly the quantity, color, and location of secretions, as well as slight fever symptoms indicated by body temperature, on the app platform. This early warning system enabled patients to increase the frequency of wound dressing changes and minimize further deterioration of wound infections. Both groups were knowledgeable about daily care and complication reporting, which is consistent with the findings by Ang et al [11] and Farrag et al [12] from interviews with 29 patients and their families on their experience with gastrostomy tubes, in which red flags for complications and seeking medical resources for tube-related issues were reported.

The experimental group experienced significantly fewer complications than the control group. Specifically, in the experimental group, there were 5 cases of complications (3 cases of infection and 2 cases of leakage) reported through daily wound checks and vital sign measurements. In comparison, the control group had 19 cases of complications (15 cases of leakage and 7 cases of infection, with 1 individual possibly experiencing 2 complications concurrently). The significant reduction in leakage complications in the experimental group can be attributed to the gastrostomy tube app platform, which primarily focused on medical care and allowed continuous uploading of daily data (wound symptoms, vital signs, complication reporting, and health care personnel's analysis of tube changes). In contrast, the control group relied on ad hoc communication to identify and report abnormalities, leading to intermittent photo uploads. The continuous uploading feature for the experimental group enabled a comparison of wound progression over time, facilitating timely detection of differences in care and adjustment of dressing change methods. Patients gained confidence in self-care through social support, changed their health behaviors, and developed a habit of daily reporting on their gastrostomy tube care. This early detection of leakage and prompt intervention helped reduce the risk of skin breakdown and physiological discomfort, such as pain. Furthermore, the lack of difference in wound infection rates between the 2 groups can be attributed to the small number of cases and the consistent use of antibiotics before and after surgery in both groups. Similarly, the lack of significant differences in emergency room visits and hospital readmissions in both groups can be attributed to the small number of cases, making it difficult to achieve statistically significant results. It is recommended that future research studies consider increasing the sample size to address this limitation.

Regarding quality of life, there was no significant difference in total scores between the experimental and control groups. This is primarily because both groups had a comparable number of patients with esophageal cancer who underwent chemotherapy or radiotherapy, which has a significant physiological and psychological impact, as highlighted by Farrag et al [12]. A series of chemotherapy and radiotherapy treatments has similar physiological effects and disease progression, with 50% of patients with head and neck tumors experiencing weight loss of 6.5% to 15% and unexpected pain after chemotherapy [12]. This is consistent with findings by Schneider et al [34], and both groups of patients experienced similar physiological challenges.

However, the experimental group had significantly higher scores in terms of quality of life at the social level than the control group. This could be attributed to the ”cost-benefit decision-making and self-efficacy in adopting healthy behaviors through the introduction of technologies such as health app usage, resulting in individual health and societal behavioral changes,” as indicated by Wong et al [27-29] with the health-social approach model and benefit of video communication. Regarding the patients’ family member experiences, they expressed that “using the gastric tube app to obtain daily information about the complications and questions and answers related to the gastric tube has been helpful and provides a sense of security.” The advantages and benefits of acquiring knowledge about gastric tube care through the app outweigh the disadvantages. Patients' changes in awareness can lead to progression to a point at which they enhance self-efficacy in gastric tube care by consistently implementing gastric tube care behaviors through regular app usage. A patient described the following:

When the tumor (cancer) was discovered, it was a sudden shock, with limited knowledge about health education and future treatment directions. Fortunately, the app helped record and take photos of the wound, etc. It helped me get through the toughest days. Recording on the app every day filled me with hope and confidence. With app management, we can discover overlooked steps and compare daily records.

This implies that, with guidance from health care professionals and through recording and quality control, patients regain confidence and self-efficacy. This is consistent with the findings by Singh et al [35] from a semistructured interview with 22 patients with spinal cord injuries, which identified the following 3 characteristics: a desire to attain health, efforts to learn and use apps, and acceptance of social support [35,36].

This study confirms the behavior change theory of the PEG app, in which patients initially set the goal of wound healing and learn about gastric tube care knowledge through the app. On a daily basis, they self-report their gastric tube health, pay attention to complications, compare changes in their gastric tube, and make timely corrections for any oversights. The social resources of feedback, guidance, and clarification from health care providers facilitate changes in patients’ self-directed health behaviors.

The lack of a significant difference in PEG knowledge between the experimental and control groups may be attributed to a couple of possible reasons. (1) Both groups had smartphones and could access PEG knowledge online and through patient support group chats, resulting in improved knowledge after intervention for both groups, and (2) both groups had instant messaging apps for immediate access to answers to questions, which could also be a contributing factor to the lack of a significant difference in knowledge. In terms of nursing application, the research findings demonstrate that the gastric tube action tracking system dynamically improved patients’ self-care knowledge, skills, and quality of life related to gastric tube management while reducing the incidence of complications and emergency department visits. Following the principle of a one-stop service, it is recommended to integrate the PEG app into the case management workflow of the hospital system, from inpatient to discharge, and expand it to cancer centers in parallel, achieving seamless integration of mHealth app information across hospital platforms, which brings substantial benefits to patients in terms of continuity of care, resulting in a win-win-win outcome. In terms of data collection, we suggest using big data approaches to collect wound evolution, vital signs, infection indicators, wound photos, and other features for early prediction of gastric tube wound infection through artificial intelligence algorithms.

### Limitations

In terms of research limitations, the participants knew to which group they belonged, which might have introduced potential biases and limitations to the objectivity of data collection, as blinding was not possible. Therefore, for future studies, it is recommended to involve personnel other than the researchers themselves in data collection to minimize researcher-related effects. With respect to research tools, considering the time and cost constraints, we suggest following the approach proposed by Cheng et al [37] to enable data exchange between the app care messages and hospital electronic medical record to efficiently transmit patients’ home-based information to the hospital, reduce unnecessary physician inquiries and ineffective examination costs, and promote the concept of remote care in smart health care. Additionally, the feature of sending reminder messages through the app required payment of SMS text messaging fees. However, due to insufficient funds, this feature did not function as intended. Instead, we resorted to using a direct phone line for notifications.

### Conclusions

The group using the PEG app indicated significant improvements in self-care abilities, reduced complications, and better social support in terms of quality of life compared with the control group. These findings highlight the benefits of the PEG app for home-based postoperative care of patients with gastrostomy tubes. This study suggests that patients discharged after their first PEG placement should be assisted in using the PEG app to facilitate independent self-management of their gastrostomy tubes at home.

## Appendix

Multimedia Appendix 1 CONSORT-eHEALTH checklist (V 1.6.1).

## Abbreviations

CVI: content validity index
mHealth: mobile health
PEG: percutaneous endoscopic gastrostomy
WHO: World Health Organization
WHOQOL-BREF: World Health Organization Quality of Life Brief Version
