# Supplementary material for: Impact of Mobile Apps in Conjunction With Percutaneous Endoscopic Gastrostomy on Patients' Complications, Quality of Life, and Health-Related Self-Care Behaviors: Randomized Clinical Trial
Source: JMIR Mhealth Uhealth. 2023 Oct 20;11:e48970. doi: 10.2196/48970 (PMC10625096; doi:10.2196/48970)
Supplement: Multimedia Appendix 1 [file mhealth_v11i1e48970_app1.pdf]

CONSORT-EHEALTH Checklist V1.6.2 Report	Manuscript Number	48970
**(based on CONSORT-EHEALTH V1.6), available at [http://tinyurl.com/consort-ehealth-v1-6].**		
**Date completed**		
10/18/2023 21:32:52		
**by**		
Li-Chan Lin		
Impact of Mobile Apps in Conjunction With Percutaneous Endoscopic Gastrostomy on Patients' Complications, Quality of Life, and Health-Related Self-Care Behaviors: Randomized Clinical Trial		
TITLE		
**1a-i) Identify the mode of delivery in the title**		
Yes, it can copy and paste from title page.		
**1a-ii) Non-web-based components or important co-interventions in title**		
**1a-iii) Primary condition or target group in the title**		
No, it is not applicable..		
ABSTRACT		
**1b-i) Key features/functionalities/components of the intervention and comparator in the METHODS section of the ABSTRACT**		
Yes, it can be copy and paste from abstract. p.1		
**1b-ii) Level of human involvement in the METHODS section of the ABSTRACT**		
**1b-iii) Open vs. closed, web-based (self-assessment) vs. face-to-face assessments in the METHODS section of the ABSTRACT**		
**1b-iv) RESULTS section in abstract must contain use data**		
**1b-v) CONCLUSIONS/DISCUSSION in abstract for negative trials**		
INTRODUCTION		
**2a-i) Problem and the type of system/solution**		
Yes, please see the manuscript p. 3-4..		
**2a-ii) Scientific background, rationale: What is known about the (type of) system**		
Yes, it can copy and paste from relevant sections.		
**Does your paper address CONSORT subitem 2b?**		
Yes, it can copy and paste from the manuscript.		
METHODS		
**3a) CONSORT: Description of trial design (such as parallel, factorial) including allocation ratio**		
Yes, it can copy and paste.		
**3b) CONSORT: Important changes to methods after trial commencement (such as eligibility criteria), with reasons**		
Yes, it can copy and paste		
**3b-i) Bug fixes, Downtimes, Content Changes**		
**4a) CONSORT: Eligibility criteria for participants**		
Yes, it can be seen in p.6.		
**4a-i) Computer / Internet literacy**		

**4a-ii) Open vs. closed, web-based vs. face-to-face assessments:**		
No, it is not necessary to explain.		
**4a-iii) Information giving during recruitment**		
**4b) CONSORT: Settings and locations where the data were collected**		
Yes, it can be seen in p.6.		
**4b-i) Report if outcomes were (self-)assessed through online questionnaires**		
Yes, it can be seen in p.6-7		
**4b-ii) Report how institutional affiliations are displayed**		
**5) CONSORT: Describe the interventions for each group with sufficient details to allow replication, including how and when they were actually administered**		
**5-i) Mention names, credential, affiliations of the developers, sponsors, and owners**		
**5-ii) Describe the history/development process**		
**5-iii) Revisions and updating**		
**5-iv) Quality assurance methods**		
**5-v) Ensure replicability by publishing the source code, and/or providing screenshots/screen-capture video, and/or providing flowcharts of the algorithms used**		
**5-vi) Digital preservation**		
**5-vii) Access**		
Yes, please see the manuscript p. 7.		
**5-viii) Mode of delivery, features/functionalities/components of the intervention and comparator, and the theoretical framework**		
Yes, it can be seen in p. 7.		
**5-ix) Describe use parameters**		
**5-x) Clarify the level of human involvement**		
**5-xi) Report any prompts/reminders used**		
Yes, it is not applicable.		
**5-xii) Describe any co-interventions (incl. training/support)**		
No, it is not applicable.		
**6a) CONSORT: Completely defined pre-specified primary and secondary outcome measures, including how and when they were assessed**		
Yes, it can be seen in p.8-9.		
**6a-i) Online questionnaires: describe if they were validated for online use and apply CHERRIES items to describe how the questionnaires were designed/deployed**		
**6a-ii) Describe whether and how "use" (including intensity of use/dosage) was defined/measured/monitored**		
**6a-iii) Describe whether, how, and when qualitative feedback from participants was obtained**		
**6b) CONSORT: Any changes to trial outcomes after the trial commenced, with reasons**		

Yes, it can be seen in p.6.		
**7a) CONSORT: How sample size was determined**		
**7a-i) Describe whether and how expected attrition was taken into account when calculating the sample size**		
**7b) CONSORT: When applicable, explanation of any interim analyses and stopping guidelines**		
Yes, it can be seen in p.8-9.		
**8a) CONSORT: Method used to generate the random allocation sequence**		
Yes, it can be seen in p.5.		
**8b) CONSORT: Type of randomisation; details of any restriction (such as blocking and block size)**		
Yes, it can be seen in p.5.		
**9) CONSORT: Mechanism used to implement the random allocation sequence (such as sequentially numbered containers), describing any steps taken to conceal the sequence until interventions were assigned**		
Yes, it can be seen in p.5.		
**10) CONSORT: Who generated the random allocation sequence, who enrolled participants, and who assigned participants to interventions**		
Yes, it can be seen in p.5.		
**11a) CONSORT: Blinding - If done, who was blinded after assignment to interventions (for example, participants, care providers, those assessing outcomes) and how**		
**11a-i) Specify who was blinded, and who wasn't**		
No, it is not applicable.		
**11a-ii) Discuss e.g., whether participants knew which intervention was the "intervention of interest" and which one was the "comparator"**		
**11b) CONSORT: If relevant, description of the similarity of interventions**		
No, it is not applicable.		
**12a) CONSORT: Statistical methods used to compare groups for primary and secondary outcomes**		
Yes, it can be seen in p.9-10.		
**12a-i) Imputation techniques to deal with attrition / missing values**		
No, it is not applicable.		
**12b) CONSORT: Methods for additional analyses, such as subgroup analyses and adjusted analyses**		
Yes, it can be seen in p.9-10.		
RESULTS		
**13a) CONSORT: For each group, the numbers of participants who were randomly assigned, received intended treatment, and were analysed for the primary outcome**		
Yes, it can be seen in p.6.		
**13b) CONSORT: For each group, losses and exclusions after randomisation, together with reasons**		
Yejs, it can be seen in p.6.		
**13b-i) Attrition diagram**		
**14a) CONSORT: Dates defining the periods of recruitment and follow-up**		
Yes, it can be seen in p.6.		
**14a-i) Indicate if critical "secular events" fell into the study period**		
**14b) CONSORT: Why the trial ended or was stopped (early)**		
Yes, it can be seen in p.6.		
**15) CONSORT: A table showing baseline demographic and clinical characteristics for each group**		
Yes, it can be seen in p.11.		
**15-i) Report demographics associated with digital divide issues**		
No, it is not applicable.		

**16a) CONSORT: For each group, number of participants (denominator) included in each analysis and whether the analysis was by original assigned groups**		
**16-i) Report multiple “denominators” and provide definitions**		
Yes, it can be seen in p.12-13.		
**16-ii) Primary analysis should be intent-to-treat**		
**17a) CONSORT: For each primary and secondary outcome, results for each group, and the estimated effect size and its precision (such as 95% confidence interval)**		
Yes, it can be seen in p.12-13.		
**17a-i) Presentation of process outcomes such as metrics of use and intensity of use**		
**17b) CONSORT: For binary outcomes, presentation of both absolute and relative effect sizes is recommended**		
Yes, it can be seen in p.12-13.		
**18) CONSORT: Results of any other analyses performed, including subgroup analyses and adjusted analyses, distinguishing pre-specified from exploratory**		
Yes, it can be seen in p.11.		
**18-i) Subgroup analysis of comparing only users**		
**19) CONSORT: All important harms or unintended effects in each group**		
No, It is not applicable.		
**19-i) Include privacy breaches, technical problems**		
**19-ii) Include qualitative feedback from participants or observations from staff/researchers**		
DISCUSSION		
**20) CONSORT: Trial limitations, addressing sources of potential bias, imprecision, multiplicity of analyses**		
**20-i) Typical limitations in ehealth trials**		
Yes, it can be seen in p.16.		
**21) CONSORT: Generalisability (external validity, applicability) of the trial findings**		
**21-i) Generalizability to other populations**		
**21-ii) Discuss if there were elements in the RCT that would be different in a routine application setting**		
**22) CONSORT: Interpretation consistent with results, balancing benefits and harms, and considering other relevant evidence**		
**22-i) Restate study questions and summarize the answers suggested by the data, starting with primary outcomes and process outcomes (use)**		
Yes, it can be seen in p.17.		
**22-ii) Highlight unanswered new questions, suggest future research**		
Other information		
**23) CONSORT: Registration number and name of trial registry**		
Yes, it can be seen in p.2.		
**24) CONSORT: Where the full trial protocol can be accessed, if available**		
Yes, it can be seen in p.7.		
**25) CONSORT: Sources of funding and other support (such as supply of drugs), role of funders**		
Yes, it can copy and paste.		
**X26-i) Comment on ethics committee approval**		

**x26-ii) Outline informed consent procedures**		
**X26-iii) Safety and security procedures**		
**X27-i) State the relation of the study team towards the system being evaluated**		
